# Adaptive Radiotherapy in Head and Neck Cancer Using Volumetric Modulated Arc Therapy

**DOI:** 10.3390/jpm12050668

**Published:** 2022-04-21

**Authors:** Nikolett Buciuman, Loredana G. Marcu

**Affiliations:** 1 Faculty of Physics, West University of Timisoara, 300223 Timisoara, Romania; bertalan_nikolett@yahoo.com; 2 OncoHelp Foundation, 300239 Timisoara, Romania; 3 Faculty of Informatics & Science, University of Oradea, 410087 Oradea, Romania; 4 Cancer Research Institute, University of South Australia, Adelaide, SA 5001, Australia

**Keywords:** planning target volume, parotid gland, recontouring, replanning, intensity modulated radiotherapy

## Abstract

A dosimetric study was performed to show the importance of adaptive radiotherapy (ART) for head and neck cancer (HNC) patients using volumetric modulated arc therapy (VMAT). A total of 13 patients with HNC who required replanning during radiotherapy were included in this study. All plans succeeded to achieve the set objectives regarding target volume coverage and organ sparing. All target volumes presented a significant decrease with an average of 76.44 cm^3^ (*p* = 0.007) for PTV_low risk_, 102.81 cm^3^ (*p* = 0.021) for PTV_intermediate risk_, and 47.10 cm^3^ (*p* = 0.003) for PTV_high risk_. Additionally, a positive correlation was found between PTV shrinkage and the number of fractions completed before replanning. Significant volume decrease was also observed for the parotid glands. The ipsilateral parotid decreased in volume by a mean of 3.75 cm^3^ (14.43%) (*p* = 0.067), while the contralateral decreased by 4.23 cm^3^ (13.23%) (*p* = 0.033). For all analyzed organs, a reduction in the final dose received after replanning was found. Our study showed that ART via rescanning, recontouring, and replanning using VMAT is essential whenever anatomical and positional variations occur. Furthermore, comparison with the literature has confirmed that ART using VMAT offers similar results to ART with intensity modulated radiotherapy.

## 1. Introduction

Radiation therapy for squamous cell carcinoma of the head and neck region often involves modern radiotherapy techniques that use modulated intensity. These techniques allow dose elevation in the tumor volume and offer a better sparing of the organs at risk (OAR), such as the parotid glands, spinal cord, brainstem, mandible, larynx, etc. Although intensity modulated arc therapy (IMRT) and volumetric modulated arc therapy (VMAT) are now standard treatments in most centers worldwide, they produce steep dose gradients, which means that any minimal changes in patient anatomy, tumor volume, and OAR position can lead to a compromised coverage of the target volume and to an overdose of critical and normal structures [1].

During radiotherapy for head and neck cancer (HNC), anatomical changes may occur starting from the first irradiation sessions, changes that include the reduction of the tumor and even normal tissues, leading to organ movement and position changes relative to other structures. Weight loss in HNC patients is also very common due to swallowing difficulties that can be caused by either the size of the tumor that prevents swallowing or by the side effects induced by radiotherapy, chemotherapy, or their combination. A quantification of weight loss among HNC patients was reported by Ho et al., showing a 7.6% drop in weight throughout treatment. In the same context, Bhandari et al. reported a 10% weight loss after the third week of radiation therapy [2,3].

The decrease of the tumor volume during HNC radiotherapy was demonstrated by Burela et al., which showed that the reduction in the planning target volume (PTV) after four weeks of radiotherapy was 13.16%, while the parotid glands decreased in volume by 27.31% and 24.63%, respectively [4]. Bhide et al. reported a reduction in the clinical target volume (CTV) after each of the first four weeks of treatment, and they concluded that the largest reduction in absolute mean volume was 21.6 cm^3^, representing 10.5% of CTV between week 0 and week 2 of radiotherapy [5].

Treatment-induced anatomical changes include, beside tumor shrinkage, normal tissue reduction and positional shift of certain structures such as the parotid glands [4]. Studies have shown that, during radiotherapy, the volume of the parotid glands can decrease by about 30%, which can lead to a shift towards the high dose region and thus overdosage. Studies have revealed that parotid gland shrinkage does not depend on the dose at treatment planning, and it indirectly reflects a higher planning dose [6,7].

Therefore, any small change in the patient’s anatomy and the tumor’s position results in significant dosimetric changes. This can lead to increased complications during treatment due to the overdose of some areas or to marginal recurrences owing to an underdose of the target volume. To minimize these effects, a possible strategy to be employed is adaptive radiation therapy (ART), which implies a CT rescanning of the patient followed by adjustments to the treatment plan, taking into account the new anatomy of the patient [4].

Most studies showing the importance of ART in HNC patients are based on the IMRT irradiation technique. In order to show the effects of ART using the VMAT technique, a retrospective study was performed at the Oncohelp Timișoara clinic, which included a total of 13 HNC patients who required replanning during treatment. Furthermore, the dosimetric outcome obtained with VMAT planning and delivery was compared to IMRT-based adaptive radiotherapy data, as reported by the literature.

## 2. Patients and Methods

### 2.1. Patient Characteristics

The study enrolled 13 non-metastatic HNC patients from the Oncohelp Timișoara clinic. The general characteristics of the patients can be found in Table 1. Eleven patients received concomitant chemotherapy during radiotherapy. A total dose of 70 Gy was administered to all patients in 2 Gy per fraction for the macroscopic disease, while the low-risk volumes received 50/54/56 Gy, delivered either by the sequential boost technique with 2 Gy per fraction, or by the simultaneous integrated boost technique with 1.6 Gy per fraction.

The simulation of the patients was performed using a Siemens CT simulator with the patients in the supine position, using, as a method of immobilization, a neck support and a custom Qfix thermoplastic mask. As per departmental protocol, 5-mm-thick slices were created and transferred to the treatment planning station (Eclipse, version 13.6) to perform VMAT contours and plans.

**Figure 1:**
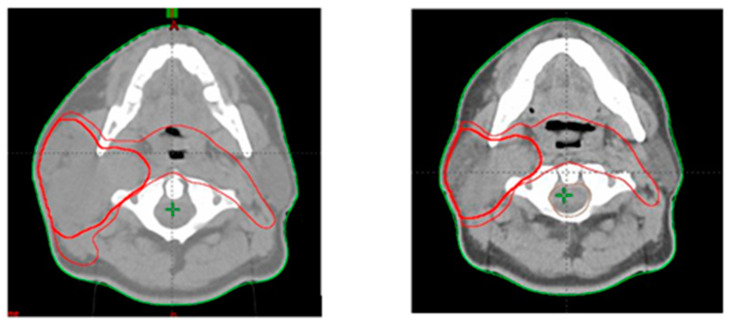
Tumor volumes contoured at first CT scan (**left**) and second CT scan after completion of 21 radiotherapy fractions (**right**).

**Figure 2:**
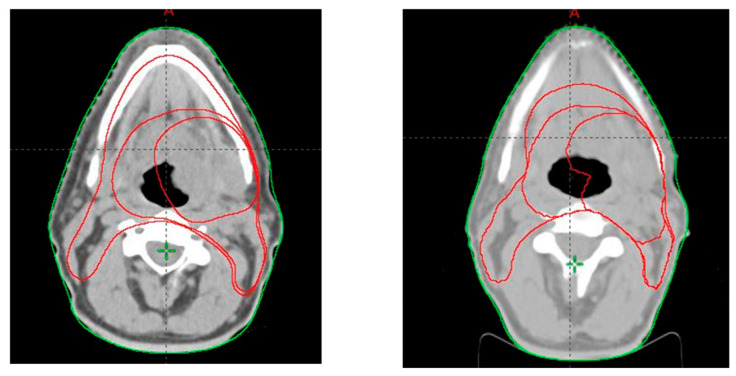
Tumor volumes contoured at first CT scan (**left**) and second CT scan after completion of 18 radiotherapy fractions (**right**).

After an average of 16 radiotherapy sessions (ranging from 3 to 25), either due to significant weight loss, visible mismatch of the immobilization mask, or a mismatch of the anatomical landmarks observed while performing imaging for daily positioning, the attending physician decided on patient rescanning and the adjustment of the treatment plan according to the patient’s new anatomy. Figure 1 and Figure 2 show the differences between tumor volumes in 2 patients who were rescanned after 21 and 18 fractions, respectively. Patients were also evaluated weekly (or more often if needed) by the radiation oncologist for monitoring treatment-related toxicity. 

### 2.2. Target Volume Delineation

For each patient, 2 or 3 planning target volumes (PTV) were outlined depending on the patient’s specific diagnosis. The PTV with the lowest dose (50/54/56 Gy), thus lowest risk of relapse, was named PTVlow and represents the 0.5 cm expansion of the CTV for the microscopic disease, the PTV with the intermediate dose (60/63/66 Gy) was labeled PTVintermediate and represents the 0.5 cm expansion of CTV with an intermediate risk of relapse (this volume was delineated only in 7 patients), and the PTV with the highest dose (70 Gy) was named PTVhigh, with the highest risk of relapse and represents the expansion with 0.5 cm of CTV for macroscopic disease. For the rescan, the same PTVs and organs at risk were delineated by the attending physician. Doses were administered either by the simultaneous integrated boost (SIB) technique or by the sequential boost technique depending on the physician’s preferences and treatment adequacy for each individual patient.

### 2.3. Treatment Planning

The initial treatment plan (called Plan1 from here onwards) was calculated for all 35 sessions that the patient intends to perform. VMAT plans were created for each patient using the objectives listed in Table 2 [8]. Planning was undertaken in two steps. The first step was to achieve PTV coverage (95% of each PTV covered by at least 95% of the prescribed dose) without neglecting the primary OAR protection constraints (parotid glands, spinal cord, and brainstem) and hotspot dose constraints (Dmax). In the second stage, an effort was made to protect the secondary OARs that appeared from case to case (eyes, lens, optic nerves, etc.) as much as possible, without compromising the stability constraints for the first step.

After performing the second CT, Plan 1 was adjusted to the number of fractions that were performed until rescan (by creating a plan revision), and a second plan (Plan 2) was made for the remaining sessions using the second CT. The final verification of the treatment plan and dose constraints was completed by summing Plan 1 with Plan 2 (PlanSum).

### 2.4. Statistical Analysis

To assess whether the replanning was justified in all cases, the values from the initial Plan 1 were compared with the summed values from the PlanSum, and the following aspects were monitored: shrinkage of the PTV, shrinkage of the parotid glands, and dose received by OARs. For this comparison, the Student’s *t*-test was used, for which we established a statistically significant value of *p* < 0.05. The Pearson correlation analysis was also completed to evaluate the association between certain variables that can affect treatment outcome.

## 3. Results

### 3.1. PTV Shrinkage

For all 13 patients evaluated in this study, two VMAT plans were created, one initial and one post-rescan. The average time after which the rescan was performed was 13 fractions. After analyzing the tumor volumes (Table 3), it was seen that, excepting for three patients, PTVlow had a significant decrease in volume with an average of approximately 76.44 cm^3^ (*p* = 0.007). Of the three patients who did not show a decrease in PTVlow, two were rescanned after 25 fractions, meaning that PTVlow was not delineated, and the volume remained the same (as a result, we eliminated them when evaluating the correlation of PTV decrease with the number of fractions after which the rescan was performed), while the other patient showed an almost insignificant volume difference of about 1 cm^3^. For PTVintermediate, in all seven patients who had this volume outlined, a significant decrease was observed with an average of 102.81 cm^3^ (*p* = 0.021). In all patients evaluated, a decrease in PTVhigh was observed on average by 47.10 cm^3^ (*p* = 0.003). 

**Figure 3:**
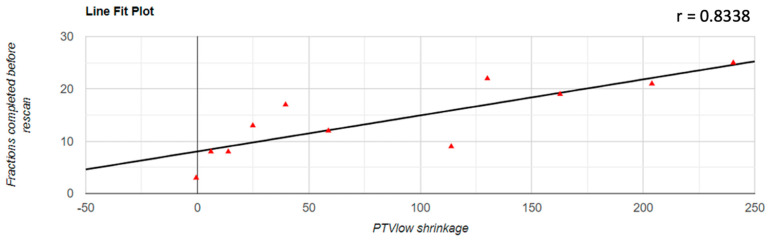
Graphical representation of the PTVlow reduction in correlation with the number of fractions completed before replanning.

**Figure 4:**
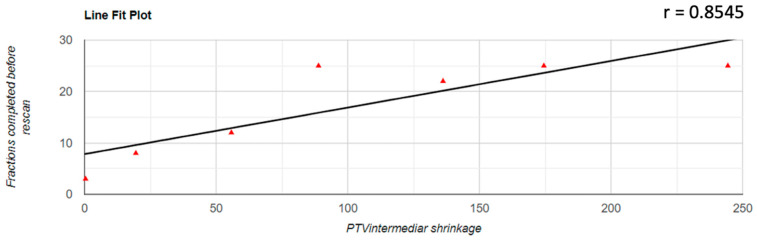
Graphical representation of the PTVintermediate reduction in correlation with the number of fractions completed before replanning.

**Figure 5:**
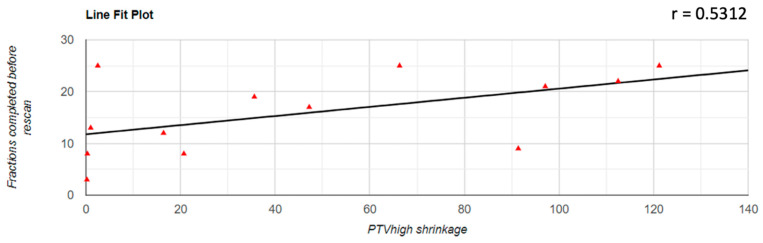
Graphical representation of the PTVhigh reduction in correlation with the number of fractions completed before replanning.

Regarding the correlation between the decrease of the PTV volume and the number of fractions completed before the replanning was performed, it was found that there was a positive correlation in all three cases; for PTVlow and PTVintermediate, this correlation was a very strong one (PTVlow: r = 0.833; PTVintermediate: r = 0.854; PTVhigh: r = 0.531). The representation of these correlations is found in Figure 3, Figure 4 and Figure 5.

### 3.2. Parotid Glands Shrinkage

Significant changes were found in the size of both the ipsilateral and the contralateral parotid. The ipsilateral parotid gland was not delineated in two cases because the tumor had a massive invasion of the gland; therefore, it could not be considered as OAR. For the ipsilateral parotid, the average decrease in volume was 3.75 cm^3^ (14.43%) (*p* = 0.067), and for the contralateral one, the average volume shrinkage was 4.23 cm^3^ (13.23%) (*p* = 0.033). The initial and after-second-scan volumes of the parotids are shown in Table 4. 

### 3.3. Dosimetry of Organs at Risk

**Figure 6:**
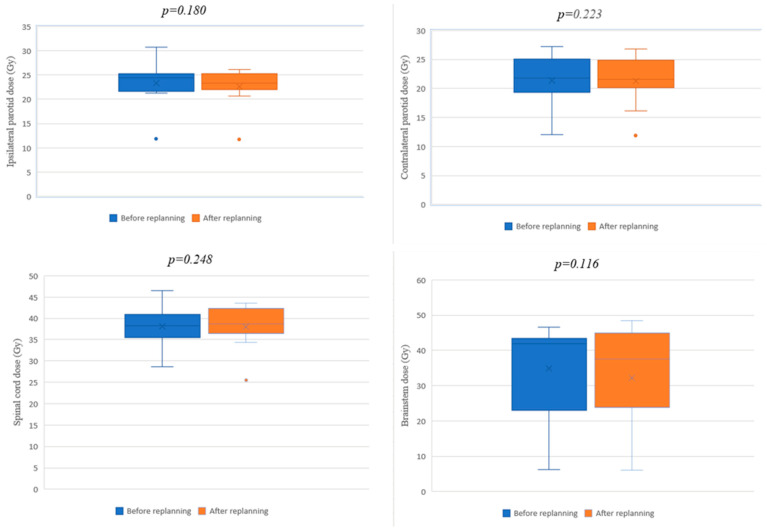
Box plot diagrams for OARs before and after replanning.

The most common organs at risk involved in HNC radiotherapy were analyzed dosimetrically, including the parotid glands, the spinal cord, and the brainstem. For all the analyzed organs, a reduction in the final dose received after replanning was found, lower than that expected at the initial scan (Table 5). Even if the values obtained after replanning were lower, these decreases were not statistically significant (ipsilateral parotid: *p* = 0.180; contralateral parotid: *p* = 0.223, spinal cord: *p* = 0.248; brainstem: *p* = 0.116). The comparative illustrations of the dosimetric differences obtained for the OARs before and after replanning are shown in the box plot diagrams (Figure 6).

### 3.4. Acute Toxicities

From the commencement of treatment until discharge, most patients developed side effects due to radiation therapy. Of the 13 patients, 69% developed grade II or III radiodermatitis, 53% of patients had grade II or grade III epithelitis, 23% of patients had radiomucositis, and 15% developed grade II xerostomia.

## 4. Discussions

The main goal of chemoradiotherapy in HNC is to improve locoregional control while maintaining the highest possible quality of life. Using modern irradiation techniques such as IMRT or VMAT, there is a need to regularly evaluate patients from several perspectives, including careful imaging evaluation before each dose fraction and ensuring the correct immobilization of the patient while confirming that the patient has not lost/gained weight and the immobilizer mask is adequately fitted.

In their review, Castelli et al. reported that after using ART, the two-year loco-regional control rates for HNC patients increased from the 80% rate that was previously reported in the literature to a rate that ranged from 88% to 97% [9]. Furthermore, daily image-guidance allowed for the possibility to reduce the planning target volume margins during intensity-modulated radiotherapy; the reduced (3 mm) CTV-to-PTV margins correlated with decreased late toxicity while providing the same locoregional control [10].

Comparing the effectiveness of ART for both IMRT and VMAT plans, Stauch et al. showed that there is no significant difference between the two techniques. However, tissue loss in HNC may have a greater effect on IMRT treatment plans when checking the plan because the number of checkpoints at which measurements are made for the plan verification is lower compared to VMAT [11]. Thomson et al. also did not find significant differences in the robustness of the plan between IMRT and VMAT in the head and neck area of patients that required replanning due to weight loss [12]. Starting from this premise, we attempted to compare the results obtained by our study with the results from the literature, whether the report results are based on IMRT plans or VMAT.

Barker et al. concluded that measurable anatomical changes that occur during radiotherapy have a dosimetric impact when highly conformal treatment techniques such as IMRT or VMAT are used. These data may therefore be useful in the development of an ART scheme that takes into account such anatomical changes and leads to an increase of the therapeutic ratio [13]. Our results showed that VMAT replanning based on repeated CT imaging was beneficial in providing adequate doses for target volumes and safe doses to normal structures for patients who underwent anatomical changes during VMAT treatment for HNC. In most cases, due to the significant decrease in target volumes, without ART, the treatment plan could lead to an overdose of the surrounding healthy tissue and an underdose of the target volume. The same results were achieved by other researchers, such as Jensen et al., who showed that, while using the IMRT technique, the coverage of the target volumes could be improved by using ART [14].

While analyzing the planning target volumes, we concluded that, for all 13 patients, rescanning was indeed necessary because the decreases in the volumes were statistically significant, on average by 76.44 cm^3^ (9.81%) for PTVlow, 102.81 cm^3^ (23%) for PTVintermediate, and 47.10 cm^3^ (23.9%) for PTVhigh. These results are supportive of other study results that showed a significant decrease of PTV during HNC radiotherapy. Hansen et al. reported a significant (7.5%) decrease of PTVlow between the first and second CT scan, whereas Zhao et al. showed an average tumor decrease of 4.14 cm^3^ after 10 fractions and 32.51 cm^3^ after 20 fractions, with an average lymph node contraction of 15.33 cm^3^ after 10 fractions and 17.27 cm^3^ after 20 fractions [15,16]. Castelli et al. showed that, at a weekly rescan, CTV70 decreased by an average of 31%, and Burela et al. obtained an average reduction of PTV of 146 cm^3^ (13.16%) after rescanning in the middle of the treatment and creating a hybrid plan [4,17]. A comparative evaluation conducted by Bhide et al. of CTV volume changes each week, as opposed to the previous week, showed that the largest reductions in the average absolute volume and the average percentage volume were 11 cm^3^ and 3.2%, respectively, between week 0 and week 2, while the absolute and percentage reductions in the following two weeks (i.e., between weeks 2 and 4) were 7 cm^3^ and 2%, respectively, values that were not statistically significant [5].

Regarding the parotid glands, we observed for the ipsilateral gland an average decrease by 3.75 cm^3^ that corresponded to a 14.43% change (*p* = 0.067) and for the contralateral parotid a decrease by 4.23 cm^3^ thus 13.23% (*p* = 0.033). Significant decreases using the IMRT technique were also reported by Burela et al., who showed a volume shrinkage by 9.84 cm^3^ (27.31%) and 8.98 cm^3^ (24.63%), respectively, for the parotid glands following their evaluation halfway through treatment [4]. Bhide et al., using the IMRT technique, observed after weekly rescanning that the largest absolute and percentage reduction in the volume of the parotid glands was 4.2 cm^3^ and 14.7%, respectively, and took place between week 0 and week 2, as in the case of the CTV volume decrease [5]. Barker et al. reported values of a median reduction in parotid volume by 0.6% per day (range, 0.2–1.8% per day), while Robar et al. observed a similar rate of shrinkage by 4.9% per week (0.7% per day) for both parotid glands [13,18]. Evaluating the parotid glands from the original scan to the sixth scan, Castelli et al. showed that the volumes of the parotid glands decreased by an average of 28.3%, similar to the data reported by others [17]. Using weekly adaptive MR-guided radiotherapy, van Timmeren et al. also showed a change in parotid and submandibular gland volume, the mean volume change being −31.9% and −29.7% after five weeks of RT [19]. The fact that parotid glands decrease in volume and shift during RT from the original position may be related to the decrease of tumor and nodal volumes, weight loss, muscle mass alteration, and changes in fat distribution, as well as fluid shift within the body [20]. The parotids medial migration towards high dose regions during the course of radiotherapy was also shown to be a source of organ shrinkage [21].

In order to evaluate the effectiveness of replanning for the risk organs, in our study, the parotid glands, the spinal cord, and the brainstem were analyzed. On average, a dose decrease was obtained for all the analyzed structures, but in each case, the differences were statistically insignificant. A study led by Bhide et al. showed that the average dose for the parotid gland increased over the study period, but the increase was not statistically significant in their case either. The differences between the maximum doses for the spinal cord and the brainstem when comparing each week’s plan with the previous week were also insignificant [5]. Thomson et al., who performed treatment rescheduling with both IMRT and VMAT, showed that there were no clinically relevant dose changes for the spinal cord and brainstem, but Dmean doses in the ipsilateral and contralateral parotid glands increased by 3.1 Gy (7.7%) and 2.5 Gy (10.4%) for IMRT plans and 3.5 Gy (8.6%) and 2.8 Gy (11.9%) for VMAT plans [12]. Castelli et al. compared the dose with and without replanning and reported that, in 85% of the plans, replanning decreased parotid Dmean by an average of 4.6 Gy [17]. Other researchers, such as Zhao et al., obtained statistically significant values for OARs and concluded that without replanning all dosimetric points for parotid gland, spinal cord, and brainstem have increased [16].

Our study has some limitations. A shortcoming is the low number of patients enrolled in this study, as well as the retrospective nature of the analysis. However, for replanning studies, the low number of patients is not uncommon, as most previous studies encompass a similar number of patients as our report [4,12,17]. Furthermore, there are other elements that can influence the interpretation of our results, such as the large variation between tumor staging (20% T4, 27% T3, 46% T2, 7% Tx) or the large differences between the number of fractions after which rescanning was performed. A number of the abovementioned studies collected their results according to a well-defined rescanning scheme such as Castelli et al. and Bhide et al., who performed weekly CT scans [5,17]. Others, such as Thomson et al., did not provide data on the number of fractions after which the rescanning took place, the need for replanning being assessed for each patient according to the pre-treatment cone beam CT evaluation [5,12,17]. It is well known that, in theory, the improvement of the delivery accuracy can be achieved by increased plan frequency; however, there are a few clinical demonstrations that show that daily plan adaptation has a major improvement in dose delivery in a clinically meaningful way. Schwartz et al. reported that by using ART for oropharyngeal squamous cell carcinoma, the majority of the dosimetric improvements from replanning can be achieved with one or two replans in the middle of the treatment [22,23]. Mid-therapy adaptive replanning in locally advanced HNC was proven effective by other studies as well, both in terms of tumor control and normal tissue toxicity [24]. The results of a clinical trial that randomized 60 HNC patients in two arms were: IMRT with replanning (arm 1) and conventional IMRT (arm 2); it showed increased complete response (96.7% in arm 1 vs. 90% in arm 2) after the six-month follow-up and decreased the dose to most organs at risk when ART was applied: spinal cord (decrease by 4.3%), ipsilateral and contralateral parotid (decrease by 6% and 2.2%) [24].

The impossibility of creating hybrid plans is also a limitation, since we could not provide any information similar to Burela et al. or Zhao et al. on the dosimetric changes (overdose or underdose in PTVs and OARs provided replanning had not taken place) [4,16]. 

Some of our patients underwent concomitant chemotherapy, which is another cause of tumor shrinkage that was not considered as an independent variable. The adaptive treatment was based on the anatomical and positional variations incurred during therapy (whether radio or chemotherapy) with the aim to counteract treatment effects through the adaptation of radiotherapy treatment planning. 

A new technique for optimizing the treatment plan at the time of delivery is available; it is online adaptive replanning (OLAR), which utilizes the imaging data acquired during daily fractions to improve the treatment plan. Nevertheless, this technique has a major drawback: the long time needed for replanning, which is important since OLAR must be accomplished online while the patient is on the couch awaiting treatment. With the improvement of auto-segmentation, contour-quality evaluation, and contour correction, the time-consuming manual review could be replaced by OLAR [25].

Most of the studies reported in the scientific literature that evaluated the impact of ART in HNC patients employed IMRT, with this technique still being the standard treatment option in a number of clinics worldwide. With the current study, our aim was to establish the role of adaptive radiotherapy via VMAT and to highlight the similar treatment facets in terms of replanning and treatment plan adaptation when compared to IMRT. Thus, VMAT can be a safe upgrade of intensity modulated radiotherapy for the management of head and neck cancer.

There are aspects of ART through the employment of advanced molecular imaging techniques that are under focus, given that tumor shrinkage often calls for the administration of higher doses for resistant volumes [26]. However, studies that attempted the administration of boosted doses to resistant tumors reported higher toxicity rates in late responding tissues, such as persistent mucosal ulcers, rendering this approach experimental [26].

Personalized treatment of cancer patients via adaptive radiotherapy is becoming a gold standard in the course of improving treatment outcomes through better tumor conformality and greater sparing of the organs at risk. A large number of centers have adopted daily verifications of tumor coverage using state-of-the-art technology, transforming image-guided radiotherapy into a routinely used technique for treatment delivery, irrespective of the tumor’s anatomical location [27,28]. Further improvements in radiotherapy will require treatment adaptation via the routine inclusion of image-guidance.

## 5. Conclusions

This study evaluated changes in target volumes and dosimetry of organs at risk in patients with HNC who received radiochemotherapy using the VMAT technique and who required adaptive radiotherapy to counteract the anatomical changes during treatment. This irradiation technique involves the use of steep dose gradients and forces the planner to minimize uncertainties (anatomical and positional variations) to maximize the therapeutic ratio. Our study showed that adaptive radiotherapy via rescanning, recontouring, and replanning using VMAT is feasible and necessary to maximize the therapeutic ratio whenever such anatomical and positional variations occur.

## Figures and Tables

**Table 1:** Patient characteristics.

Sex	Age (Years)	Tumor Location	Staging	Concomitant Chemotherapy
M	57	Pyriform sinus	T4N2bM0	Yes
M	68	Larynx	T2N2M0	Yes
M	48	Rhinopharynx	TxN3M0	Yes
M	50	Rhinopharynx	T2N3M0	Yes
M	33	Nasopharynx	T3N2M0	No
F	68	Rhinopharynx	T4cN2M0	No
F	55	Rhinopharynx	T2N2M0	Yes
F	62	Tongue	T3N3M0	Yes
M	52	Rhinopharynx	T3N2M0	No
M	50	Rhinopharynx	T2N0M0	Yes
M	76	Hypopharynx	T2N2M0	Yes
M	65	Hypopharynx	T4N3M0	No
M	47	Rhinopharynx	T2N1M0	Yes

**Table 2:** Dose constraints used to create the treatment plan.

Organ	Dose-Volume Index	Objective
PTV	D95%	V95%
	Dmax	<108%
Parotid glands (single)	Dmean	<26 Gy
	V50%	<30 Gy
Spinal cord	Dmax	<45 Gy
Brainstem	Dmax	<54 Gy

**Table 3:** The size of the PTVs at the first and second scan.

PTV Low (cm^3^)		PTV Intermediate (cm^3^)		PTV High (cm^3^)		Fractions Completed before Rescanning
Initial	Replanning	Initial	Replanning	Initial	Replanning	
587.9	574	-	-	72.1	71.8	8
577.9	571.8	360.2	340.7	204	183.3	8
1032.8	828.9	-	-	317.6	220.5	21
1028.8	898.7	896	759.8	300	187.5	22
881.9	719.2	-	-	229.1	193.5	19
871.2	757.3	-	-	489.2	397.8	9
764.4	764.4	342.2	253.3	159.8	93.5	25
586.4	527.6	236.8	181	50.7	34.3	12
1058.9	1058.9	711.4	467	80	77.5	25
726.4	701.5	-	-	171	170	13
479.8	480.3	164.6	164.2	34.3	34.1	3
634.6	595	-	-	231.9	184.7	17
904.4	664	390	215.5	165	43.8	25
Mean Value						
779.646	703.2	443.028	340.214	192.669	145.562	
*p* Value						
0.007		0.021		0.003		

**Table 4:** Ipsilateral and contralateral parotid size at first and second scan.

Ipsilateral Parotid (cm^3^)	Ipsilateral Parotid (Replanning) (cm^3^)	Contralateral Parotid (cm^3^)	Contralateral Parotid (Replanning) (cm^3^)
32	32.7	25.3	28.9
19.5	18.8	14.6	14.5
-	-	43.1	26.8
30.7	31.9	26.1	25.5
26.1	18.7	23.1	18.3
-	-	17.6	24.6
36.8	33.4	33.4	24
10	16.5	18.9	12.3
35.5	22.2	34.2	14.5
18.2	18	18.3	17.6
26	27.2	29.2	28.7
18.7	13.8	12.5	12.5
32.3	18.4	27.8	20.9
Mean Value			
25.98	22.23	24.93	20.7
*p* Value			
0.067		0.033	

**Table 5:** Dosimetry of organs at risk before and after replanning.

Ipsilateral Parotid (Gy)	Ipsilateral Parotid Replan (Gy)	Contralateral Parotid (Gy)	Contralateral Parotid Replan (Gy)	Spinal Cord (Gy)	Spinal Cord Replan (Gy)	Brainstem (Gy)	Brainstem Replan (Gy)
11.826	11.719	12.03	11.912	28.597	25.508	11.34	12.178
21.599	20.61	16.285	16.144	34.380	34.301	6.284	6.149
-	-	20.907	21.297	40.905	42.26	42.974	46.24
22.169	23.33	19.273	20.658	40.042	40.873	44.675	48.464
25.327	25.558	25.058	24.869	42.072	42.881	43.4	44.91
-	-	23.373	23.476	36.936	38.685	23.007	23.908
24.449	22.862	20.663	20.127	38.33	36.427	42.685	25.035
21.224	23.337	21.808	21.577	38.496	37.518	41.847	24.242
24.714	26.09	25.066	26.791	37.506	37.486	41.345	40.916
27.419	25.239	27.222	24.991	46.546	43.62	46.641	44.17
25.061	24.282	23.44	22.621	35.491	38.641	40.03	37.63
21.958	21.946	21.140	22.084	35.851	36.217	31.922	30.953
30.687	23.632	18.935	13.486	42.210	37.26	55.44	55.133
Mean Value							
23.312	22.600	21.169	20.771	38.258	37.821	36.276	33.840
*p* Value							
0.180		0.223		0.248		0.116	
